# Purification of Hydrogen from CO with Cu/ZSM-5 Adsorbents

**DOI:** 10.3390/molecules27010096

**Published:** 2021-12-24

**Authors:** Mihail Mihaylov, Elena Ivanova, Videlina Zdravkova, Stanislava Andonova, Nikola Drenchev, Kristina Chakarova, Radoslav Kefirov, Rositsa Kukeva, Radostina Stoyanova, Konstantin Hadjiivanov

**Affiliations:** Institute of General and Inorganic Chemistry, Bulgarian Academy of Sciences, 1113 Sofia, Bulgaria; eivanova@svr.igic.bas.bg (E.I.); videlina@svr.igic.bas.bg (V.Z.); s.andonova@svr.igic.bas.bg (S.A.); ndrenchev@svr.igic.bas.bg (N.D.); kristina@svr.igic.bas.bg (K.C.); r.kefirov@svr.igic.bas.bg (R.K.); rositsakukeva@yahoo.com (R.K.); radstoy@svr.igic.bas.bg (R.S.)

**Keywords:** adsorption, breakthrough curves, carbon monoxide, Cu/ZSM-5, FTIR spectroscopy, hydrogen purification, temperature-programmed desorption

## Abstract

The transition to a hydrogen economy requires the development of cost-effective methods for purifying hydrogen from CO. In this study, we explore the possibilities of Cu/ZSM-5 as an adsorbent for this purpose. Samples obtained by cation exchange from aqueous solution (AE) and solid-state exchange with CuCl (SE) were characterized by in situ EPR and FTIR, H_2_-TPR, CO-TPD, etc. The AE samples possess mainly isolated Cu^2+^ cations not adsorbing CO. Reduction generates Cu^+^ sites demonstrating different affinity to CO, with the strongest centres desorbing CO at about 350 °C. The SE samples have about twice higher Cu/Al ratios, as one H^+^ is exchanged with one Cu^+^ cation. Although some of the introduced Cu^+^ sites are oxidized to Cu^2+^ upon contact with air, they easily recover their original oxidation state after thermal treatment in vacuum or under inert gas stream. In addition, these Cu^+^ centres regenerate at relatively low temperatures. It is important that water does not block the CO adsorption sites because of the formation of Cu^+^(CO)(H_2_O)_x_ complexes. Dynamic adsorption studies show that Cu/ZSM-5 selectively adsorbs CO in the presence of hydrogen. The results indicate that the SE samples are very perspective materials for purification of H_2_ from CO.

## 1. Introduction

One of the strategic priorities of the future is the transition to a hydrogen economy. Hydrogen is the most “environmentally friendly” fuel, as its combustion produces only water that does not pollute the environment. Therefore, hydrogen is considered the main fuel of the future, with a wide range of applications: stationary and mobile.

Significant difficulties in some practical applications of hydrogen are related to its purification and storage. Hydrogen liquefies at 20.3 K, so efforts are focused on the development of hydrogen storage materials [1,2]. The purity of hydrogen is an essential requirement not only for its effective sorption but also for its subsequent use. For example, in order to prevent catalyst poisoning in fuel cells, it is necessary that the CO content in hydrogen does not exceed 10 ppm [3,4]. Therefore, hydrogen purification is an important technological step before its storage. Pressure swing adsorption (PSA) is used to effectively purify H_2_ from CO_2_ [5,6,7,8,9], and catalytic oxidation is used to remove oxygen impurities [10]. In order to purify from CO, catalysts for the preferential oxidation of CO in the presence of H_2_ have been developed [11,12]. Membranes are often used to remove various gas impurities, while metal hydrides are effective in separating H_2_ from inert components [4,6,13]. 

Due to their high energy efficiency and the ability to achieve high selectivity in the separation of gases, adsorption technologies are increasingly attractive [14]. In order for an adsorbent to be effective in the purification of hydrogen from CO, the enthalpy of adsorption of the two gases must be very different. While, as a rule, hydrogen adsorption is weak in most cases, there are ample possibilities to control the enthalpy of CO adsorption. This is due to the possibility of forming a back π-bond with the adsorption centres, as well as to the synergistic effect between σ- and π-bonds [15,16]. Significant additional stabilization of the adsorption forms of CO can also be achieved with cations with high coordinative unsaturation, such as cations exchanged in zeolites [15,16,17,18]. Indeed, it has been theoretically predicted [19] that the difference in the adsorption enthalpy of H_2_ and CO on cation-exchanged zeolites is sufficient to allow the separation of the two gases. This theoretical prediction needs experimental confirmation.

The aim of the present work is to study the possibilities for purification of hydrogen from CO with Cu/ZSM-5 zeolites. Copper was chosen as the active component because the monovalent state is typical of it, thus allowing the formation of stable carbonyls as well as because of its relatively low price. Indeed, copper-modified MOF was recently proposed for the separation of CO and H_2_ [20]. However, we have chosen ZSM-5 as a support material as it can stabilize active metal centres both in a low oxidation and low coordination state [15,21,22,23,24,25], which favour CO adsorption. The effects of the exchange and activation procedures were studied by various methods (IR spectroscopy of adsorbed molecules, TPR, TPD, EPR, UV-VIS spectroscopy). It has been shown that Cu^+^ ions can play the role of selective adsorption sites for CO in the presence of hydrogen, oxygen, and water vapor. The method of ion exchange makes it possible to control the amount and energy of the adsorption centres. In this respect, the solid-state exchange is superior to the conventional one (from aqueous solution) as it allows for a ca. twice as high concentration of the centres, at the same time being easier to regenerate.

## 2. Results

### 2.1. Initial Characterization of the Samples

#### 2.1.1. Chemical Composition

The main zeolite that was used in this study originated from Zeolist. For comparison purposes, a Degussa H-ZSM-5 sample was also used. This material and the respective Cu-containing sample were in particular suitable for the IR studies because of the low level of spectral noise. Some characteristics of the two H-ZSM-5 samples are presented in Table 1.

The Cu/ZSM-5 samples were obtained by conventional ion exchange in aqueous solution (AE) and solid-state cation exchange (SE). The notation of the sample and the concentration of copper are presented in Table 2.

Although the conditions of solid-state exchange suggest the removal of excess CuCl, the data from the analysis show a certain content of chlorine in the samples (see Table S1 and Figure S1 from the Supporting Information). Photometric analysis indicated 1.5 and 1.35 wt. % chlorine for the 9.7CuZ23(SE) and 6.4CuZ27(SE) samples, respectively. This chlorine is probably mainly in the form of small amounts of unreacted CuCl occluded in the pores of the zeolite, although the possibility of other forms cannot be ruled out. Thus, the data indicate that up to 28% of copper in 9.7CuZ23(SE) is in the form of CuCl and the respective value for 6.4CuZ27(SE) is 38%.

#### 2.1.2. Phase Composition and Morphological Characteristics

**Figure 1:**
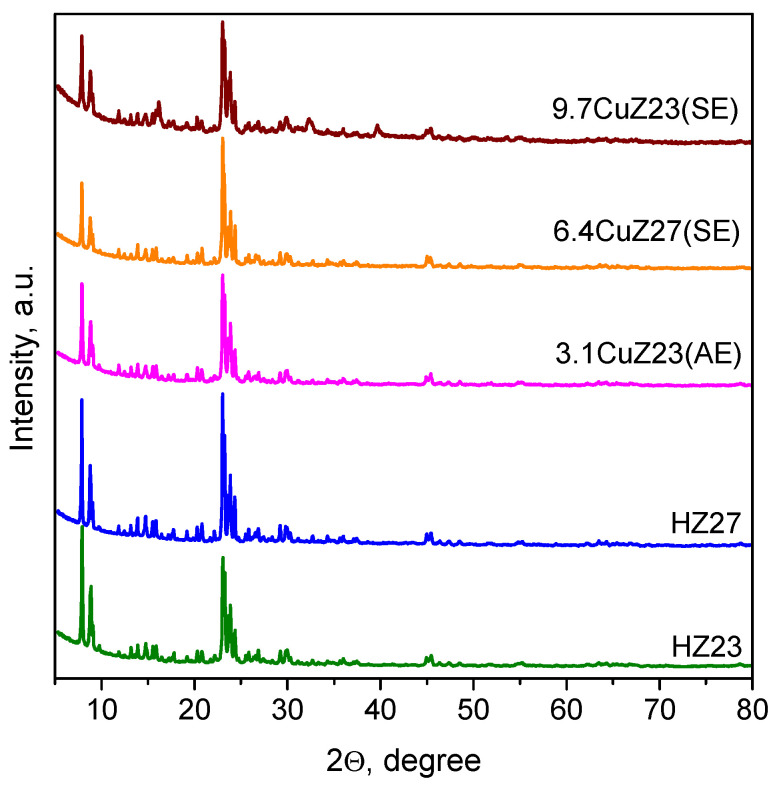
XRD profiles of the parent and the Cu-exchanged zeolites.

XRD analysis confirmed that the starting ZSM-5 zeolites possessed an MFI structure (Figure 1). The crystallite size, determined by the Sherrer equation, is 40 nm for HZ23 and 80 nm for HZ27.

After the ion exchanges, the materials retain the size and morphology of their particles. No new phases were observed by XRD in the cation-exchanged zeolites. Only traces of CuAlCl_4_ (2Θ = 16.24°) were detected with the 9.7CuZ23(SE) sample (see Figure 1). Therefore, any metal oxides and/or chlorides eventually present should be amorphous and highly dispersed in the zeolite matrix. We also detected small expansion in the cell parameters (by 0.1–0.3%) related to the introduction of copper into the zeolite matrix. 

#### 2.1.3. Textural Characteristics

The starting H-ZSM-5 zeolites possess a developed specific surface area (Table 1). The specific surface area of the zeolites exchanged in the aqueous medium (Table 2) is slightly reduced, which indicates successful modification with atomically dispersed cations, while maintaining the microporous structure. The solid-phase exchange leads to a more significant reduction in the specific surface area. This is probably due to the partial blocking of the pores with oxide and/or chloride clusters.

#### 2.1.4. In Situ FTIR Spectroscopy in the OH Stretching Region

**Figure 2:**
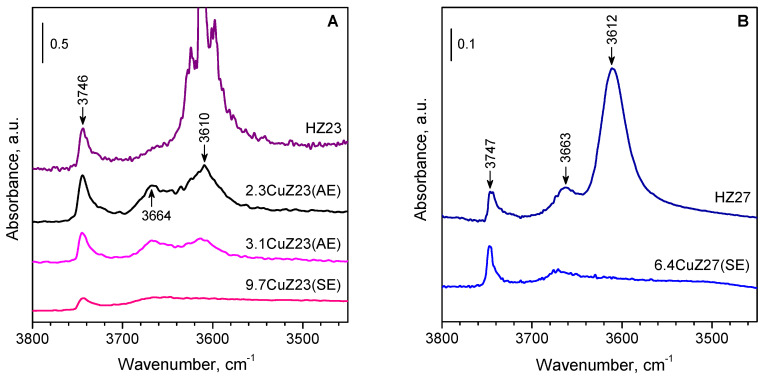
(**A**,**B**) IR spectra in the ν(OH) region of the parent and the Cu-exchanged zeolites. All samples were preliminary evacuated at 400 °C.

IR spectroscopy was used to monitor the proton exchange. After the introduction of copper, all zeolites show a loss of intensity for the band of the acidic bridging hydroxyl groups, which can serve as a measure of the degree of cation exchange [26,27,28,29]. Figure 2 shows the IR spectra of the parent zeolites and the cation-exchanges samples in the ν(OH) region. 

The observed decrease in intensity for the band at 3610 cm^−1^, which is characteristic of Brønsted acid sites, agrees well with the values for the exchange degree calculated on the basis of the chemical analysis. The band of the bridging hydroxyls was practically absent from the spectrum of the CuZ(SE) samples. The band at 3740 cm^−1^, characteristic of Si-OH groups, is hardly affected after cation exchange. However, some decrease was observed for the band at 3660 cm^−1^ due to aluminol groups.

#### 2.1.5. XPS

**Figure 3:**
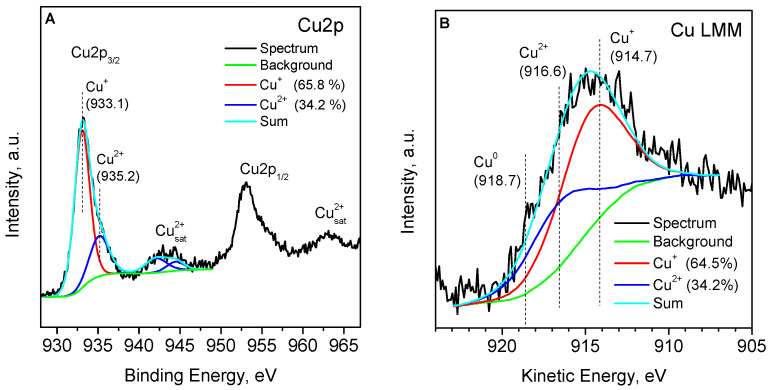
XPS spectrum of Cu 2p (**A**) and Cu LMM (**B**) for the 6.4CuZ27(SE) sample.

The 6.4CuZ27(SE) sample was examined ex situ with XPS. XPS analysis of the Cu 2p peaks can distinguish Cu^+^ and Cu^2+^ cations based on the value of the binding energy and the presence of satellite peaks, typical of divalent cations [30,31,32,33,34,35,36,37]. The Cu 2p_3/2_ core level observed in this case can be deconvoluted into two Gaussian curves with maxima at a binding energy of 933.1 and 935.2 eV, which correspond to Cu^+^ and Cu^2+^ species (Figure 3A). When performing deconvolution, the area ratio of the Cu^2+^ peak and its satellite of about 1.7 is fixed, which guarantees the correct differentiation of Cu^2+^ from Cu^+^ [33]. Thus, about 65% of copper is in the form of Cu^+^-isolated cations and possibly small amounts of CuCl and Cu_2_O. The results show that part of the copper is oxidized to Cu^2+^ upon exposure of the sample to air. These considerations are also supported by the Auger spectrum (Figure 3B), which shows that the kinetic energies of the sample correspond to a mixed state of Cu^2+^ and Cu^+^ on the surface [35]. According to Bolis et al. [32], residual CuCl (partially oxidized by air to CuCl_2_) acts as a protective cap, hindering the oxidation of exchanged Cu^+^ inside the pores of the zeolite. 

#### 2.1.6. EPR 

**Figure 4:**
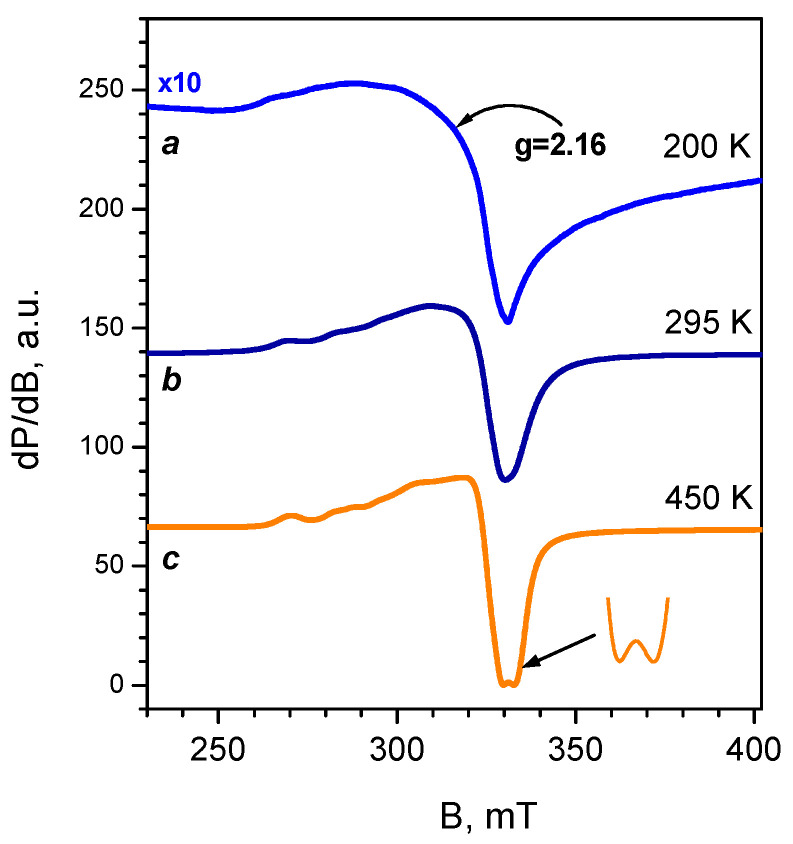
In situ EPR spectra of 2.3CuZ23(AE) annealed in O_2_ at 400 °C recorded at different temperatures.

**Figure 5:**
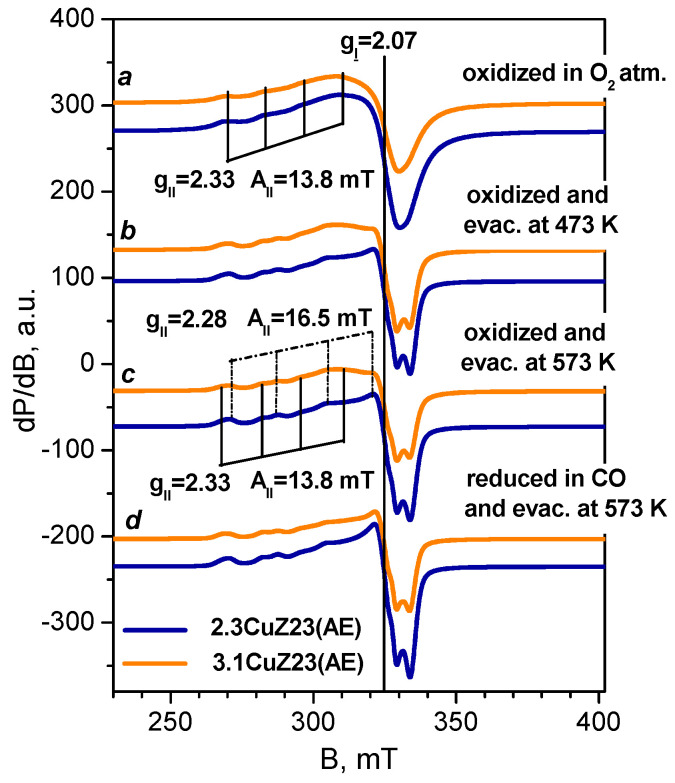
In situ EPR spectra at 295 K of 3.1CuZ23(AE) and 2.3CuZ23(AE): annealed in O_2_ at 400 °C (**a**), evacuated at 200 °C (**b**), evacuated at 300 °C (**c**) and reduced with CO at 300 °C (**d**). The simulated EPR parameters are designated for selected samples.

EPR spectroscopic analysis provides valuable information on the coordination state and the number of isolated Cu^2+^ cations in the zeolites, while the associated Cu^2+^ ions remain “invisible” by this technique [38,39,40,41]. The resistance of the isolated Cu^2+^ ions to reduction conditions was checked in order to evaluate the amount of inactive copper. Two samples were studied by EPR, namely those obtained by liquid phase exchange and expected to possess a significant concentration of isolated Cu^2+^ sites. The two samples have virtually identical EPR spectra. In order to dehydrate the sample whilst avoiding auto-reduction, they were pre-treated in a vacuum and then in oxygen at a high temperature then cooled in the presence of oxygen. EPR spectra of 2.3CuZ23(AE) recorded in situ at 295 K consist of a single signal with axial symmetry (Figure 4, spectrum b) with the following parameters determined after simulation: g_II_ = 2.33; g_⊥_ = 2.07; A_II_ = 13.8 mT. The simulated spectrum is shown in Figure 5, spectrum a. When the spectra are recorded at a lower temperature (200 K), this signal decreases in intensity and a broad isotropic signal with g ≈ 2.16 appears (Figure 4, spectrum a). Recording the spectra at a higher temperature (450 K) leads to an increase in intensity as well as to signal splitting (Figure 4, spectrum c). The new signal has g_II_ = 2.28 and A_II_ = 16.5 mT. A similar spectrum was observed after thermo-vacuum treatment of the samples (Figure 5, spectra b–c). The presence of two signals is attributed to Cu^2+^ cations with different coordination to the oxygen atoms of the zeolite [39,40,41]. The observed changes in the spectrum with increasing temperature are most likely related to the removal of water molecules from the coordination sphere of the Cu^2+^ sites and a decrease in its symmetry [41].

In order to study the stability of the isolated Cu^2+^ ions under reduction conditions, EPR spectra were registered in situ after evacuation at different temperatures and after reduction with CO (Figure 5). The parameters determined after simulation are shown for selected spectra. Evaluation of the number of spins based on the EPR and chemical analysis showed that the samples contain mainly isolated Cu^2+^ ions, so any existing pairs are expected to be few in number. The comparable (within the experimental error) content of isolated Cu^2+^ ions in the two samples agrees with the expected increase in the number of associated Cu^2+^ ions with increasing copper content in the zeolite and with the data from H_2_-TPR studies (see below). Evacuation at 300 °C leads to a decrease in the concentration of isolated Cu^2+^ cations because of their auto-reduction. An additional decrease was observed after reduction with CO at the same temperature. Although isolated cations are thought to be more difficult to reduce than associated ones, the results demonstrate the possibility of CO reduction and even auto-reduction of some isolated cations.

#### 2.1.7. DR UV-Vis Spectroscopy

**Figure 6:**
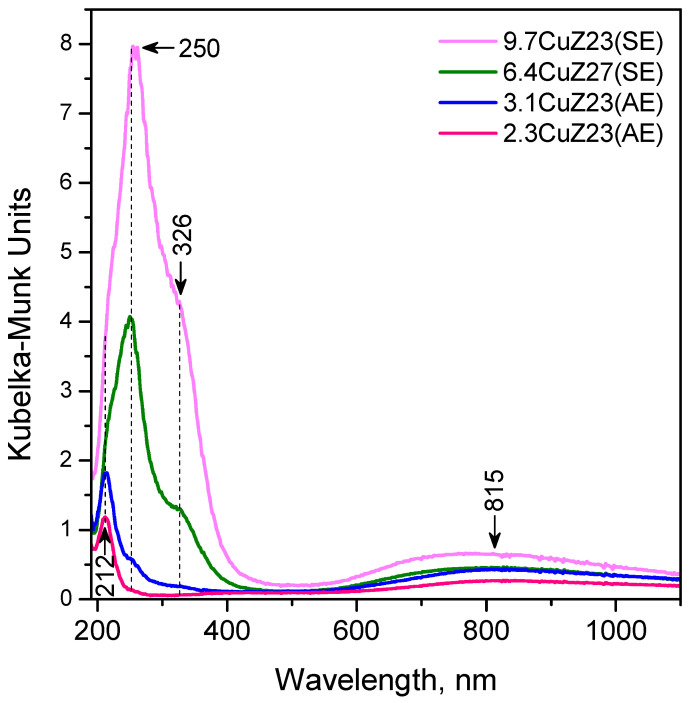
DR UV-Vis spectra of the Cu-exchanged zeolites.

Hydrated copper-exchanged zeolites (exposed to air) have a pale green colour with the intensity increasing with the copper content. In their diffuse reflectance, UV-Vis spectra bands were observed at 212, 250, 326, and ~800 nm (Figure 6). The broad absorption band at about 800 nm is attributed to the 2E_g_ → 2T_g_ d-d transitions in distorted octahedral aqua complexes of Cu^2+^ [40,42,43,44]. No CuO phase is registered in the structure of which the copper cations are nearly in T_d_ environment and are characterized by d-d transitions in the region above 1000 nm [43,44]. The two bands at 212 and 250 nm are attributed to a low-energy O^2−^ → Cu^2+^ ligand-to-metal charge transfer (LMCT) between lattice oxygen and isolated Cu^2+^ ions located in an asymmetric octahedral environment (with two short and two long O···Cu bonds) [44]. The band at 326 nm is associated with copper-oxide structures with a chain-like and square-planar coordination of extra-lattice oxygen ligands, located in zeolite channels [43,44]. No bands due to Cu dimers at 360–455 nm [43,45] were observed in this work. Finally, it seems that the majority of Cu^+^ ions exchanged from gas phase are reoxidized to Cu^2+^ upon contact with air, which is in line with the XPS results. 

#### 2.1.8. TPR

**Figure 7:**
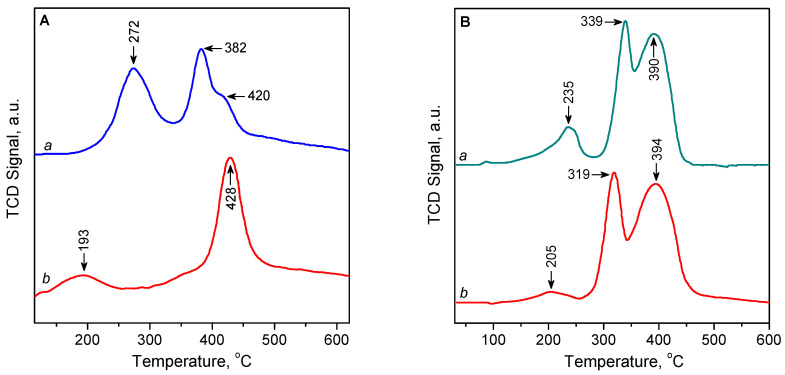
(**A**) H_2_-TPR profiles of 3.1CuZ23(AE): pre-treated in He/O_2_ at 500 °C (a) and reduced with He/CO at 300 °C (b). (**B**) H_2_-TPR profiles of 9.7CuZ23(SE): pre-treated in He at 500 °C (a) and reduced with He/CO at 300 °C (b).

The H_2_-TPR profile of the oxidized 3.1CuZ23(AE) sample shows two main maxima, at 274 and 382/420 °C (Figure 7A, curve a), which are attributed to a successive reduction in isolated Cu^2+^ ions, first to Cu^+^ and then to Cu^0^ [29,37,42,46,47,48,49,50,51,52,53]. The H_2_-TPR profile of the sample pre-reduced with CO is different (Figure 7A, curve b). In this case, the two maxima occur at ca. 195 and 430 °C, with the first peak appearing with a significantly reduced intensity and making up about 10% of the total area of the peaks. The peak at 430 °C is attributed to the reduction of Cu^+^ cations obtained by the preliminary reduction with CO. The peak at 195 °C is associated with Cu^2+^ species residual to reduction with CO [46]. These results show that only a small fraction of Cu^2+^ centres remain unreduced in the CO stream and therefore will not play a role in the CO adsorption during separation. Similar results were obtained for the 2.3CuZ(SE) sample (Figure S2 from the Supporting Information); however, in this case, the proportion of the isolated Cu^2+^ sites resisting reduction with CO is slightly higher, ~15%. This is consistent with the results of in situ EPR and CO-TPD studies. 

The amount of H_2_ consumed for the reduction of the sample 3.1CuZ23(AE) (expressed as H_2_/Cu molar ratio) is summarized in Table S2. The total amount of H_2_ consumed by oxidized 3.1CuZ23(AE) corresponds to a ratio of H_2_/Cu = 0.97, which shows that virtually all Cu^2+^ ions have undergone a two-electron reduction to Cu^0^. The amount of H_2_ consumed is almost equally distributed between the reduction peak 1 (0.51 H_2_/Cu) and reduction peaks 2 and 3 (0.46 H_2_/Cu). This suggests that most of the copper species had been in oxidation state 2+ and were completely converted to Cu^+^ during the reduction corresponding to peak 1 and to Cu^0^ during the reduction at higher temperature (peaks 2 and 3). The area of peak 1 is about 10% larger than that of peaks 2 and 3, i.e., about 5% of Cu^2+^ (probably in the form of dispersed CuO [35]) is directly reduced to Cu^0^. After pre-treatment with CO, H_2_ consumption is almost half, as most Cu^2+^ are already reduced to Cu^+^. 

As in the solid-state exchanged samples the initial state of copper was in the form of Cu^+^ ions, they were activated in an inert (not oxidizing) atmosphere before the H_2_-TPR experiments. XPS and UV-Vis studies have shown that exposure to air leads to partial oxidation of Cu^+^. Therefore, the samples were also examined after reduction with CO to check whether the latter leads to the generation of additional Cu^+^ centres. The TPR curve of the sample activated in inert atmosphere showed the presence of three peaks at 235, 339, and 390 °C (Figure 7B, curve a). The first low-intensity peak can be attributed to the reduction of copper oxide or isolated Cu^2+^ centres to Cu^+^, while those at higher temperatures can be attributed to the reduction of Cu^+^ cations [29]. The TPR profile of the CO-reduced sample has a similar shape and area (Figure 7B, curve b). This indicates that the reduction with CO does not generate a significant additional amount of Cu^+^ ions from what has already been obtained by auto-reduction when heated in a stream of inert gas. The maxima are slightly shifted and are observed at 205, 319, and 394 °C. The intensity of the low-temperature peak decreased significantly (by 85%), as copper oxides had already been reduced by CO. It is possible that the peak at 205 °C is due to isolated Cu^2+^ cations, which cannot be reduced by CO. A similar picture was observed for 6.4CuZ27(SE) (Figure S3 from the Supporting Information).

The H_2_/Cu ratio during the reduction of activated 9.7CuZ23(SE) is = 0.67, which corresponds to a ratio of Cu^2+^:Cu^+^ = 25:75 and is in line with the result of XPS (Table S3). Peak 1 is most likely due to the reduction of small amounts of dispersed CuO [35,37]. The high hydrogen consumption for peaks 2 and 3 (H_2_/Cu ratio above 0.5) indicates that about 12% of them are associated with the direct reduction of Cu^2+^ species to metal. These results can be explained by the presence of small amounts of Cu^2+^ species that are occluded before the reduction of some Cu^+^ species. This supposition is consistent with the high copper loading in the SE samples. After the reduction of the Cu^+^ species and formation of metal particles, the occluded Cu^2+^ species become accessible and their reduction contributes to peaks 2 and 3. With the sample preliminary reduced by CO, the Cu^2+^: Cu^+^ ratio decreases to 16:84 only at the expense of the drop of the area of peak 1, which is consistent with the hypothesis of the presence of occluded Cu^2+^ species.

### 2.2. FTIR Spectroscopy of Adsorbed CO

CO adsorption was studied by in situ IR spectroscopy. CO is the most widely used probe molecule, which can provide detailed information about adsorption centres. In particular, there are a number of studies on the adsorption of CO on Cu-ZSM-5 and the formation of mono and di-carbonyls of Cu^+^ ions and, at low temperatures, tri-carbonyl, has been well established [15,23,30,32,54,55,56,57,58]. The possibility of formation of polycarbonyls is related to the high coordinative unsaturation of the Cu^+^ sites in the zeolite, which determines their high electrophilicity and high stability of the carbonyl complexes formed. 

**Figure 8:**
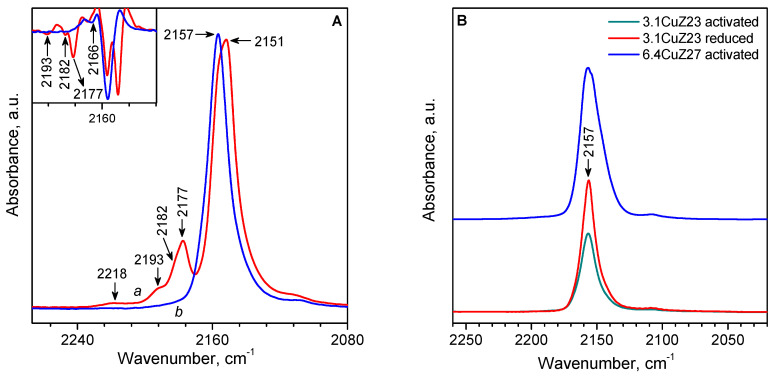
(**A**) IR spectra of CO adsorbed on activated 3.1CuZ23(AE): 10 mbar CO at room temperature (a) and after evacuation at room temperature (b). (**B**) IR spectra of carbonyl complexes on different samples stable after evacuation at room temperature.

In the IR spectrum of CO adsorbed at room temperature and under equilibrium pressure on activated 3.1CuZ23(AE), bands typical of Cu^+^(CO)_2_ dicarbonyls (2177 and 2151 cm^−1^) are observed, as well as low-intensity bands characterizing small amounts of Cu^+^(CO)_3_ tricarbonyls (2193 and 2168 cm^−1^) (Figure 8A). Since Cu^2+^ cations do not adsorb CO under these conditions, the results show that the thermovacuum treatment resulted in a partial autoreduction of Cu^2+^ to Cu^+^. After evacuation at room temperature, the polycarbonyls are converted to stable Cu^+^-CO monocarbonyls (2157 cm^−1^) The observed additional bands in some of the spectra (di-carbonyls at 2182 cm^−1^ and monocarbonyls at 2166 cm^−1^) indicate some heterogeneity of the Cu^+^ cations, which is consistent with the results from EPR and CO-TPD (see below).

Comparison of activated and reduced 3.1CuZ23(AE) shows that reduction with CO increases the concentration of Cu^+^ in the zeolite by about 50%, i.e., about two-thirds of the reducible Cu^2+^ are auto-reduced under vacuum, and CO is required to reduce the remaining third. Some authors believe that only copper from [Cu-O-Cu]^2+^ pairs is reduced under a vacuum. Our results indicate that some isolated Cu^2+^ may also be auto reduced.

Unlike the conventional ion-exchange method, the solid-state cation exchange allows twice as many copper atoms to be introduced into the zeolite, directly in the state of isolated Cu^+^ cations [29]. Indeed, the 6.4CuZ27(SE) sample showed a significant capacity for CO even after thermovacuum activation. Upon subsequent reduction with CO, the number of active Cu^+^ sites remains virtually unchanged, as evidenced by the negligible changes in the IR spectrum of adsorbed CO. In accordance with the higher copper content in sample 6.4CuZ27(SE), the band of Cu^+^-CO complexes is more intense compared to sample 3.1CuZ23(AE) (Figure 8B). Here again, heterogeneity of the Cu^+^ sites was observed, as well as a second type of Cu^+^(CO)_3_ (2188 cm^−1^), which was not observed for 3.1CuZ23(AE). The monocarbonyls on 6.4CuZ27(SE) decompose completely under a dynamic vacuum at about 250 °C.

It should be noted that the principal Cu^+^-CO complexes are observed at the same frequency, 2157 cm^−1^, although according to TPD data, these complexes in CuZ(SE) are more unstable than those for conventionally exchanged CuZ(AE) samples. This is in line with reports in the literature [59], according to which ν(CO) is not sensitive to the binding energy of CO in these complexes.

IR spectroscopic studies were also performed to elucidate the effect of possible impurities of water vapor and oxygen on CO adsorption in the 6.4CuZ27(SE) sample. In accordance with our earlier work on Cu-ZSM-5, the coadsorption of CO and water leads to the formation of mixed-ligand Cu^+^(CO)(H_2_O)x (x = 1, 2) complexes [15,55,60]. Upon evacuation, these complexes lose their H_2_O ligands stepwise, and the recovered monocarbonyl complexes remain thermally stable. Similar to these results, it was found that the Cu^+^ ions in the 6.4CuZ27(SE) sample remain active with respect to CO adsorption, even in the presence of certain amounts of moisture (see below). 

Additional studies have shown that Cu^+^-CO species remain unchanged after staying for 20 h in an O_2_ atmosphere (50 mbar), i.e., the formation of carbonyls stabilizes the monovanent oxidation state of copper. The concentration of the Cu^+^-CO species decreases only after heating in oxygen at temperatures above 150 °C. The relatively high adsorption capacity of CuZ(SE) relative to tightly bound CO, with simultaneous tolerance to moisture and oxygen, give a preliminary indication of the potential importance of this material as an adsorbent for CO capture.

Finally, we note that no adsorption forms of H_2_ were detected on the samples at room temperature. 

### 2.3. TPD of CO

**Figure 9:**
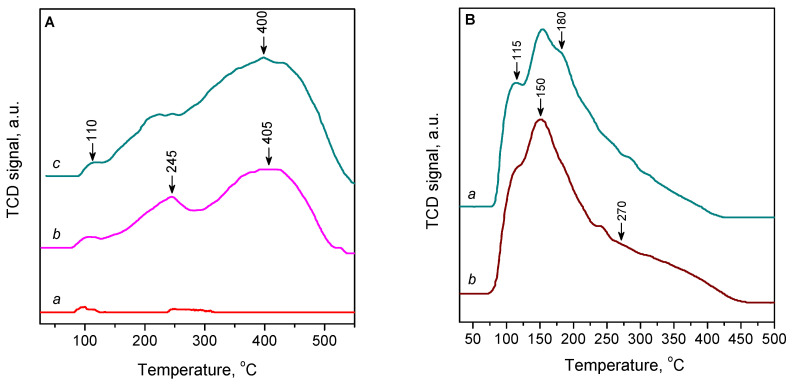
(**A**) CO-TPD curves of 2.3CuZ23(AE) activated in O_2_/He (a) and samples reduced with CO/He: 2.3CuZ23(AE) (b) and 3.1CuZ23(AE) (c). (**B**) CO-TPD curves of sample 9.7CuZ23(SE) activated in He (a) and reduced with CO/He (b).

The results of CO-TPD studies confirm that Cu^2+^ ions in ZSM-5 zeolite do not adsorb CO at an ambient temperature. Indeed, in the CO-TPD curve for the oxygen-activated 2.3CuZ23(AE) zeolite, no desorption of CO was practically recorded (Figure 9A, curve a). However, when the sample was preliminarily reduced with CO, a significant amount of desorbed CO was observed, as most of the Cu^2+^ ions were converted to Cu^+^ sites strongly adsorbing CO (Figure 9A, curve b). Two main TPD maxima were observed, at 245 and 405 °C. These peaks are associated with desorption of CO from Cu^+^ cations at different positions: located on the channel walls and on the intersections, respectively [59,61,62].

A similar CO-TPD curve was also observed for reduced 3.1CuZ23(AE) (Figure 9B, curve c). In this case, however, the desorbed amount of CO is higher. The sorbent efficiency, expressed by the CO/Cu ratio, was higher, 0.90 for 3.1CuZ23(AE) versus 0.75 for 2.3CuZ23(AE) (Table 3). This result is explained by the increased proportion of reducible Cu^2+^ cations. The reducibility is probably related to the localization of the copper sites and their participation in oxygen–cation pairs.

For the CuZ(SE) samples, significant CO desorption is observed even when they are not subjected to pre-reduction with CO. The reason for this is that air-oxidized cations (formed according to XPS and UV-Vis spectroscopy data) easily regain their initial oxidation state of +1 when heated in a stream of inert gas. In the CO-TPD curve for 9.7CuZ23(SE), a desorption maximum was observed at 150 °C with shoulders at 115 and 180 °C and a broad feature at about 270 °C (Figure 9B). We observed similar results for sample 6.4CuZ27(SE) (Figure S4, Supporting Information). The analogous TPD profile was also reported for Cu-ZSM-5 prepared by solid-state exchange and ascribed to CO desorption from exchanged Cu^+^ ions with an average Cu-O coordination number of ~2.7 [63]. The positions of these maxima clearly show that the SE method gives a different distribution of Cu^+^ centres in the zeolite matrix as compared to the conventional AE method. In this case, the directly introduced Cu^+^ sites by the SE method adsorb CO less strongly and can thus be regenerated more easily. 

The effect of temperature on adsorption can be estimated from the TPD curves [64]. The area under the TPD profile is proportional to the adsorbed amount, while the maximum TPD peak depends on the strength of the adsorption. The TPD studies have shown that SE samples are characterized by high capacity and relatively weak adsorption centers, which, on the one hand, makes them more sensitive to elevated temperatures, but, on the other hand, can be considered as an advantage in terms of the energy required to regenerate the adsorbent.

We investigated the effect of adsorption temperature (T_ads_) on the amount of CO chemisorbed on the 9.7CuZ23(SE) sample (Figure S5.). The stepwise increase in the adsorption temperature (25, 55, 78, and 103 °C) leads to a gradual decrease in the adsorbed amount (20, 15, 11, and 8 mL g^−1^, respectively), by about 25–28% for a 25 °C increase in T_ads_. However, the results show that, even at 100 °C, the sample retains a significant adsorption capacity.

The larger area of the CO-TPD curve for the CuZ(SE) samples as compared to CuZ(AE) is related to the larger amount of exchanged copper ions. However, it appears that only part of the copper centres in the CuZ(SE) samples (~55%) “work” in the adsorption of CO. The inaccessibility of some of the centres is probably related to blocked access to them and existence of bulk copper-containing phases. Note, however, that the CuZ(SE) samples do not need CO-reduction to be effective. 

In order to evaluate the contribution of zeolite and possible impurities of copper oxides in the adsorption of CO, mixtures of 15% CuO/HZ27 and 15% Cu_2_O/HZ27 (mechanically mixed and ground in an agate mortar) were studied. In the CO-TPD curves of these samples, low-intensity peaks were observed at about 110, 220, and 240 °C, respectively. Thus, it seems that TPD-peaks of CO possibly bound to copper oxides, at 220 and 240 °C [65], are masked in the TPD curves of the Cu-ZSM-5 samples, and the peak at 110–120 °C is probably due to the occlusion of CO in the pores of the zeolite (confirmed by experiment with the parent H-zeolites). Another experiment with a mechanical mixture of 15% CuCl/HZ27 showed that CuCl desorbs CO at about 360 °C (weak peak). Therefore, it is possible that the high temperature peak observed in the CO-TPD curves of CuZ(SE) could be at least partly due to the desorption of CO from residual CuCl.

### 2.4. Dynamic Adsorption Measurements

In order to estimate the ability of the investigated metal-exchanged zeolites to capture CO from hydrogen gas streams, their adsorption properties were studied in dynamic mode. Measuring the adsorption of gases in such a way makes it possible to assess the effectiveness of adsorbents when separating gases under conditions close to industrial [6,66,67].

**Figure 10:**
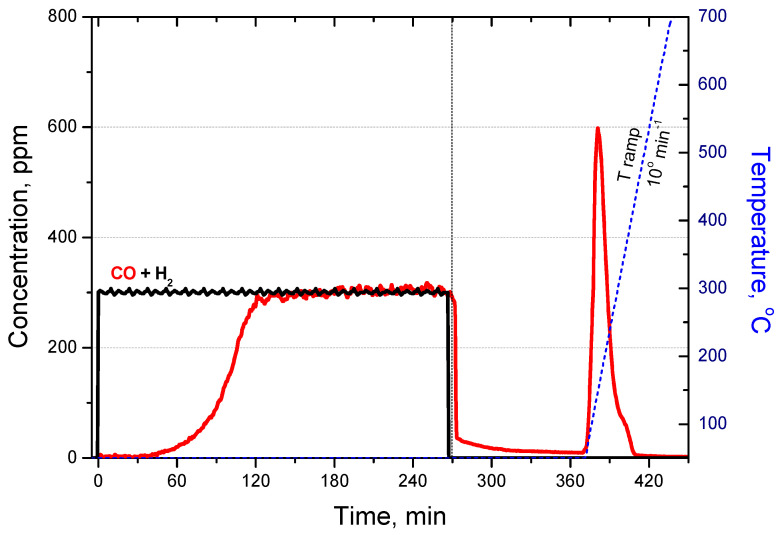
Breakthrough curves for binary gas mixture of CO + H_2_ on 9.7CuZ23(SE) followed by subsequent TPD.

As an example, Figure 10 shows the experimental breakthrough behaviour of binary gas mixtures of CO + H_2_ and the subsequently registered TPD profile. The breakthrough curves clearly show that the column holds CO (impurity), while hydrogen (purified gas) passes unhindered through the adsorbent layer. This is also confirmed by the fact that only CO is registered during the TPD. The reason for this is that the Cu^+^ centres in zeolite bind CO in stable complexes, while their interaction with H_2_ is very weak. Preliminary studies have shown that the presence of hydrogen in the stream does not affect the saturation curves of CO. Therefore, further concentration profiles for H_2_ are not presented in the figures. 

#### 2.4.1. Effect of Different Factors on the Breakthrough Curves

A series of experiments were initially conducted to elucidate the effect of various factors on the CO breakthrough curves and to identify the optimal experimental conditions. In addition, the behaviour of CuZ(SE) adsorbents was investigated in the presence of oxygen or water impurities.

**Figure 11:**
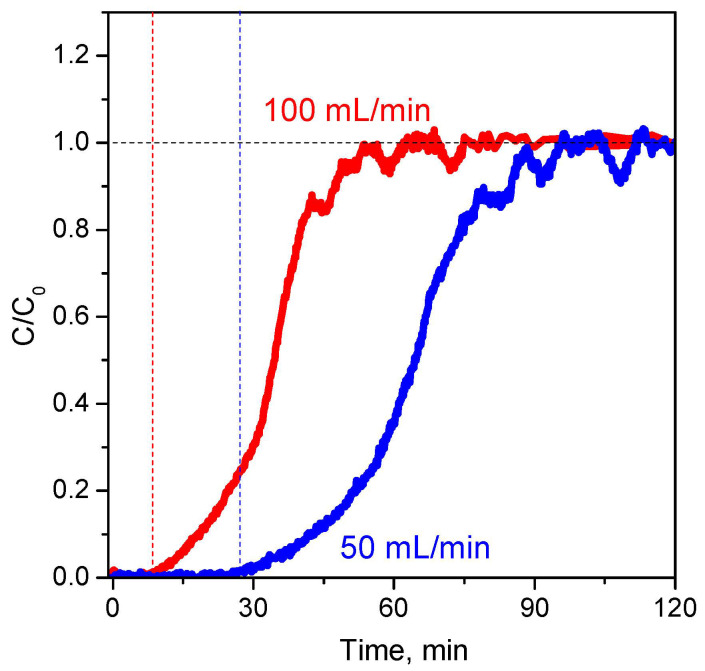
Breakthrough curve of CO on 6.4CuZ27(SE) at flow rates of 50 and 100 mL min^−1^.

(1) Effect of the gas flow. The increase in the flow rate of 50 to 100 mL min^−1^ leads to a faster saturation of the 6.4CuZ27(SE) sample with CO (Figure 11). In addition, the breakthrough curve is steeper, which is an indication of a faster sorption kinetics. Due to the lower adsorption capacity of AE samples, the dynamic sorption studies were carried out at the lower flow rate, 50 mL min^−1^. This allowed registration of the saturation zone in the breakdown curves for these materials and comparison of their adsorption characteristics with those of the SE samples. 

(2) Effect of adsorbate concentration. Increase in the adsorbate concentration has a similar effect as increasing the flow rate. As the CO concentration increased from 200 to 300 ppm, the breakthrough time for the 6.4CuZ27(SE) layer decreased, and the mass transfer zone (MTZ) became narrower, and the height of the unused bed (HUB) became lower (Figure S6A, Supporting Information). For reasons similar to the above described, the lower adsorbate concentration was selected for routine experiments from these studies. 

(3) Effect of oxygen. The addition of O_2_ (700 ppm) to the stream had a negligible effect on the CO breakthrough curve for the 9.7CuZ23(SE) sample (Figure S6B, Supporting Information). This shows that the active Cu^+^ sites introduced into the zeolite by solid-state exchange are stable in an oxidizing atmosphere and “work” in the presence of oxygen impurities. 

(4) Effect of humidity. It was found that in the case of traces of water vapour, the twice-higher water content in the feed stream has practically no effect on the breakthrough curve of CO on 6.4CuZ27(SE) and, respectively, on the sorption capacity (Figure S7A, Supporting Information). The amount of water deposited on the sample was assessed on the basis of the amount of water released in a subsequent TPD experiment (Figure S7B, Supporting Information). 

#### 2.4.2. CO Breakthrough Curves

Breakthrough curves of CO were measured for different samples in the presence of H_2_ under the same dynamic conditions. The following important characteristics of the adsorbent can be obtained from the breakthrough curves [66]: capacity at full saturation (capacity at full equilibration), denoted by CO_ads_^e^. This is the amount of adsorbate removed by unit weight of the adsorbent at the saturation point (equilibration time) at which the entire bed is in equilibrium with the feed and the effluent concentration reaches the feed value (i.e., C/C_0_ = 1). The amount of adsorbate adsorbed at any given time is related to the area above the breakthrough curve at that time, bounded by the line C/C_0_ = 1; the breakthrough capacity (working capacity), denoted by CO_ads_^b^, is defined as the amount of sorbate removed by the sorbent bed at break point time (t_b_), when the concentration of adsorbate leaving the bed increases to the minimum detectable concentration. The breakthrough point can be defined also as the point at which the effluent concentration begins to increase rapidly; bed utilization efficiency, defined as the breakthrough capacity divided by the capacity at full saturation; HUB, height of the unused bed at breakthrough (in this work the total bed height was 5 mm). The data obtained are summarized in Table 4.

Oxidized 3.1CuZ23(AE) adsorbs negligible amounts of CO (Figure S8, Supporting Information). The saturation capacity is about 1.4 mL g^−1^, and the breakthrough (working) is practically null. This is consistent with the results of IR and TPD CO adsorption studies, which showed that Cu^2+^ sites in zeolite do not adsorb CO. The small amount of adsorbed CO is probably related to a limited reduction of some Cu^2+^ to Cu^+^. Indeed, after reduction with CO, the adsorption capacity strongly increased (saturation capacity of ~9.8 mL g^−1^ and working capacity of 3.9 mL g^−1^). In this case, the results showed that the reduction with CO plays a crucial role in the activation of the adsorbent. Desorption measurements showed that the majority of the centres were regenerated at a relatively high temperature (~390 °C). 

The CuZ(SE) samples were also examined (Figure S9, Supporting Information). These samples showed the highest breakthrough capacities, and no preliminary reduction with CO is necessary. The 6.4CuZ27(SE) sample had the full equilibration capacity of ~14 mL g^−1^ and the capacity at break point of ~6 mL g^−1^. The respective values determined for the 9.7CuZ23(SE) sample were ~26 and 11 mL g^−1^. CO was removed from the SE samples at a much lower temperature as compared to the AE sample.

Finally, several adsorption–desorption cycles were performed on 9.7CuZ23(SE) to study both the adsorption reproducibility and the cyclic stability of the adsorbent. Figure S10 from the Supporting Information demonstrates the unchanging behavior of the 9.7CuZ27(SE) adsorbent in three successive adsorption/desorption cycles. This is a good indication of the possible practical application of the material.

## 3. Discussion

In the present work, the possibilities of using Cu/ZSM-5 as an adsorbent for purification of hydrogen from CO have been investigated. Two groups of samples were studied—prepared by aqueous exchange (AE) and by solid state exchange (SE). The results obtained show that all Cu/ZSM-5 samples could be used as a material for purification of hydrogen from CO and the active sites for the process are Cu^+^ cations. As hydrogen is adsorbed relatively poorly, it does not interfere with CO adsorption. However, the SE samples demonstrate some important advantages. 

Furthermore, we have shown that the synthesis method has a significant effect on both the adsorption capacity and the energy characteristics of the adsorption. In the samples obtained by AE, the initial oxidation state of copper is 2+ and these Cu^2+^ ions do not adsorb CO at room temperature, i.e., the as-synthesized materials are not applicable as an adsorbent for the intended purpose. However, after reduction, Cu^+^ ions are generated, which adsorb strongly CO. 

The initial oxidation state of copper in the samples obtained by SE is 1+. Although some oxidation of Cu^+^ to Cu^2+^ occurs after exposure of the samples to air, the initial copper oxidation state is easily recovered even after activation under an inert gas stream. Moreover, additional reduction has little effect on the amount of Cu^+^ cations. In these cases, the regeneration of the adsorbent is greatly facilitated because of the weaker Cu^+^-CO bond. The IR spectra in the hydroxyl region confirmed that all acidic protons were exchanged by copper cations. In this method of synthesis, a more significant decrease in the specific surface of the zeolite was observed, which was attributed to the blockade of the pores by oxide and/or chloride clusters. However, the samples obtained by SE appear more convenient from a practical point of view: they show a high adsorption capacity, and no preliminary reduction treatment is necessary.

It should be noted that practically no difference in the breakthrough behaviour of the CuZ(SE) adsorbents was observed after pre-activation in an inert gas Ar or a reducing gas mixture, CO/Ar. This not only confirms the expected stabilization of monovalent metal cations by the zeolite matrix but indicates that the adsorption centres are in active form after activation in an inert gas and do not need to be generated by CO reduction prior to the gas separation process. The results of subsequent TPD treatment also showed that these adsorbents can be recovered after CO saturation at relatively low temperatures, which would save energy costs for their regeneration. At the same time, the materials keep a high adsorption capacity up to ca. 100 °C. In addition, the adsorbents are stable in several subsequent adsorption/desorption cycles, which is important for their practical applications. 

It has been reported [59,61] that the Cu^+^ ions from the walls of the MFI channels are coordinated to three or four oxygen atoms from six-membered rings, while those at the intersection of the channels, to two oxygen atoms from an AlO_4_ tetrahedron. It is believed that after adsorption of CO, the copper cations are coordinated to two zeolite oxygens. In the first case, Cu ions are drawn from the walls of the channels, and the bonds with the oxygen atoms that do not belong to AlO_4_ tetrahedra are broken. Upon desorption, the initial copper coordination is restored. Due to the energy gain from the back coordination of Cu^+^ to additional oxygen atoms, these cations lose their CO ligands at lower temperatures. We found that the distribution of Cu^+^ ions in the zeolite depends on the mode of cation exchange. SE favours the occupation of the channel wall positions and, thus, the formation of weaker adsorption sites.

An important peculiarity of the samples studied is that they can operate even in the presence of water vapour and oxygen. It is known that Cu^+^ cations in Cu^+^-ZSM-5 can for mixed ligand Cu^+^(CO)(H_2_O)_x_ complexes [15,55,60], which is the reason for the applicability of these materials as CO adsorbents in the presence of water. On the contrary, one could expect that O_2_ could oxidize Cu^+^ cations to Cu^2+^ and thus fade the adsorption capacity of the material towards CO. However, it appears that the oxidation process is slow, and the material keeps (or slightly loses) its capacity. 

The evaluation of the efficiency of CuZ(SE) adsorbents showed that more than half of the deposited copper was active in CO adsorption. At the same time, the height of the unused layer at the time of breakthrough is large. A possible reason for this is the occlusion of cuprous oxide and/or cuprous chloride in the zeolite pores. In support of this conclusion, a relatively wide MTZ was observed, which is an indication of difficult mass transfer as a result of blocking the pores from these possible impurities. Further work is necessary to establish the effect of the residual chlorine: on the one hand, it seems that it reduces the number of “working” copper sites, but it is also possible for the chlorine to affect the strength of CO adsorption. 

The main conclusion of this work is that, from the two groups of materials studied, the CuZ(SE) samples demonstrate the higher potential as adsorbents for CO capture in the presence of hydrogen, both in terms of capacity and from a techno-economic point of view related to their regeneration. In addition, the separation of CO is not affected by the presence of impurities of oxygen or moisture.

Optimizing the synthesis by different ways (using nano-zeolites or zeolites with mesopores, changing the Si/Al ratio in ZSM-5, taking care of more complete chlorine removal, etc.) would allow additional optimization of the adsorbents.

## 4. Materials and Methods

### 4.1. Synthesis of the Samples

A NH_4_-ZSM-5 zeolite was purchased from Zeolist. In order to convert it to the hydrogen form, the material was heated at 500 °C, first in a stream of air and then in a stream of inert gas. Another zeolite, H-ZSM-5, was supplied by Degussa. Some characteristics of the H-forms of zeolites are presented in Table 1.

The Cu/ZSM-5 samples were obtained by cation exchange, performed by two different methods: (i) conventional exchange from aqueous solution (AE) and (ii) solid-state exchange (SE). 

In the conventional cation exchange [54,68,69], a certain amount of the starting zeolite is suspended in a reflux flask containing an aqueous solution of Cu(CH_3_COO)_2_·xH_2_O. The suspension is stirred with a magnetic stirrer at 50 °C for 2 h then filtered, dried, and calcined in air at 500 °C. 

The 6.4CuZ27(SE) sample was synthesized by solid-state exchange [23,29,63]. The annealed, dehydrated HZ27 was mixed with CuCl (supplied in an ampoule) in a molar ratio of Cu/Al = 1. The mixture was ground in an agate mortar and then transferred to a quartz reactor. A stream of helium was fed to the reactor at a flow rate of 50 mL min^−1^ and the temperature was raised at a rate of 2 K min^−1^ to 750 °C. The mixture was heated at this temperature for 40 h and then cooled to room temperature. The 9.7CuZ23(SE) sample was prepared again by solid-state exchange, but the CuCl + HZ23 mixture (CuCl being in small excess) was subjected to thermovacuum treatment in a glass reactor connected to a vacuum system: first at 200 °C (2 h) to remove any traces of moisture and then at 300 °C for 20 h. At this temperature, CuCl sublimates and penetrates the pores of the zeolite. Finally, the product was heated at 500 °C to remove excess CuCl. Solid-state exchange requires the application of special measures to exclude the access of moisture during the synthesis procedure. For this purpose, the whole procedure of mixing the zeolite with the salt and transferring it to the reactors is carried out in a glove box filled with inert gas.

### 4.2. Chemical Analysis

X-ray fluorescence (XRF) analysis was used to estimate the composition of samples. It was performed using a Total reflection X-ray fluorescence spectrometry analyser S2 PICOFOX (Bruker AXS GmbH, Karlsruhe, Germany). 

The content of aluminum and copper was determined by ICP analysis. The ICP OES measurements were performed with a Jobin Yvon ICP spectrometer (JY ULTIMA 24, Horiba Advanced Techno, Co., Ltd., Kyoto, Japan) with plasma generator frequency 40.67 MHz, equipped with a cyclonic spray chamber and a concentric nebulizer. 

The chlorine content in the samples obtained by exchange with CuCl was measured spectrophotometrically (portable NOVA, Merck KGaA, Darmstadt, Germany) using the respective Spectroquant^®^ tests (Merck).

### 4.3. Nitrogen Physisorption

Brunauer–Emmett–Teller (BET) surface areas were determined by low-temperature (77.4 K) nitrogen adsorption using NOVA 1200e apparatus (Quantachrome Instruments, Boynton Beach, FL, USA). The samples were degassed at 573 K under a vacuum to ensure a clean dry surface.

### 4.4. FTIR Spectroscopy

The FTIR spectra were recorded with Thermo Scientific Nicolet 6700 (Thermo Fisher Scientific Co., Madison, WI, USA) and Nicolet Avatar 360 (Nicolet Co., Madison, WI, USA) FTIR spectrometers accumulating 64 scans at a spectral resolution of 2 cm^−1^. Self-supporting pellets (ca. 10 mg cm^−2^) were prepared from the sample powders and treated directly in a purpose-built IR cell allowing measurement at ambient and low temperatures. The IR cell was connected to a vacuum-adsorption apparatus with a residual pressure below 10^−4^ Pa. Before adsorption, CO (Merck, 99.5%) and H_2_ (Messer, 99.9999%) were additionally purified by passing through a liquid nitrogen trap. 

### 4.5. XRD

Powder X-ray diffraction (XRD) analysis was performed with a Bruker D8 Advance diffractometer with a CuKα radiation and a LynxEye solid position-sensitive detector (Bruker AXS Advanced X-ray Solutions GmbH, Billerica, MA, USA). XRD patterns were recorded in the range of 5.3 to 80° 2θ with a constant step of 0.02° 2θ and counting time of 17.5 s per step. The mean crystallite size was determined by Topas-4.2 software.

### 4.6. XPS

The XPS measurements were carried out on an AXIS Supra electron spectrometer (Kratos Analytical Ltd., Manchester, UK) using achromatic AlKα radiation with a photon energy of 1486.6 eV. The energy calibration was performed by normalizing the C_1s_ line of adsorbed adventitious hydrocarbons to 284.6 eV. The binding energies (BE) were determined with an accuracy of ±0.1 eV. The deconvolution of the peaks was performed using the commercial data-processing software ESCApeTM from Kratos Analytical Ltd.

### 4.7. EPR

The EPR spectra of Cu-containing zeolites were recorded as the first derivative of the absorption signal of a EMXplus EPR spectrometer (Bruker, Karlsruhe, Germany) in the X-band (9.4 GHz). A variable temperature unit ER4141VTM was used for temperature variation. The EPR spectra were simulated by the program SimFonia (Bruker) and the quantitative analysis was performed by the licensed program SpinCount (Bruker).

### 4.8. DR UV-Vis Spectroscopy

The DR UV–VIS spectra were taken with a Thermo Scientific Evolution 300 spectrophotometer (Thermo Scientific Co., UK) equipped with a Praying Mantis Diffuse Reflectance Accessory.

### 4.9. Thermo-Programmed Studies

Temperature programmed reduction (TPR) and temperature programmed desortion were carried out using ChemBET TPR/TPD apparatus (Quantachrome Instruments, Boynton Beach, FL, USA) equipped with a thermal conductivity detector.

For TPR, the samples (~80 mg) were reduced in a 10% H_2_/Ar with a flow rate of 20 mL min^−1^. The temperature was increased from room temperature to 973 K at a ramping rate of 10 K min^−1^. Before the TPR, the samples were heated to 773 K in a flowing stream of He for SE samples or of 5% O_2_/He for AE zeolites as well as after reduction in 5% CO/He flow at 300 °C for 30 min.

The TPD procedure included three stages: activation, adsorption, and thermodesorption. In the first stage, the activation of the samples was performed in three possible modes: (i) oxidative—O_2_/He at 500 °C, (ii) neutral—He at 500 °C, or (iii) reduction—CO/He at 300 °C. In the second stage, a stream of inert gas containing CO (5%) passes through the layer of adsorbent until the adsorption capacity is saturated. In the third stage, the TPD profile of desorption was registered, i.e., the amount of CO desorbed in a He stream (20 mL min^−1^) was monitored with a linear increase in temperature (10 K min^−1^). The amount of desorbed CO was determined on the basis of an injected dose of 0.1 mL CO or CO obtained by a thermal decomposition of about 2 mg CaC_2_O_4_·H_2_O (Alfa Aesar, 99.9985%).

### 4.10. Breakthrough Curve Mesurments

The breakthrough curves were measured on a CATLAB system Hiden Analytical (Warrington, UK) equipped with an HPR-20 R&D mass spectrometer. The gas stream was passed at a flow rate of 50 mL/min through a ~30 °C thermostated adsorption column filled with zeolite (50 mg, granules 0.25–0.4 mm) in a fixed bed (column diameter D = 7 mm, total bed height H_T_ = 5 mm). The feed gas stream contains, in addition to argon (carrier gas), typically 300 ppm CO and 300 ppm hydrogen. In some experiments, the concentrations were 200 ppm, and this is specially noted. The adsorbate concentration in the column effluent was analysed by mass spectrometer. After recording the breakthrough curve, thermoprogrammed desorption (TPD) is performed to determine the optimum temperature for adsorbent regeneration. In this case, a stream of argon with a flow rate of 20 mL/min was passed through the column, and the temperature was raised linearly at a rate of 10 °C min^−1^ from 50 to 600 °C. Prior to each experiment, the samples were activated (regenerated) in a gas stream (50 mL/min) with a defined composition and a linear increase in temperature (10 °C min^−1^) to a specified value: (i) 4% O_2_/Ar (500 °C, 5 min), (ii) Ar (500 °C, 5 min), or (iii) 0.4% CO/Ar (300 °C, 30 min). In some cases, several adsorption–desorption cycles were performed to study both the reproducibility of adsorption and the stability of the adsorbent and to assess the influence of various factors on the process (bed height, flow rate, adsorbate concentration, presence of H_2_, presence of oxygen or moisture impurities).

## 5. Conclusions

Cu/ZSM-5 zeolites obtained by solid-state exchange using CuCl are perspective materials for the purification of H_2_ from CO. Carbon monoxide is much more strongly adsorbed on the Cu^+^ sites than H_2_. The adsorption process occurs even in the presence of O_2_ and H_2_O in the gas phase. The synthesis procedure based on solid-state exchange ensures a high concentration of Cu^+^ sites, and it was found that they are more easily regenerated as compared to the Cu^+^ sites produced on Cu-ZSM-5 prepared by conventional ion-exchange from aqueous solutions. Although the dynamic adsorption measurements show a good performance of the samples, there are possibilities of additional optimization of the adsorbent nature/composition, e.g., change in the Si:Al ratio in the parent ZSM-5 materials, particle size, use of other zeolites and/or removal of residual chlorine species.

## Figures and Tables

**Table 1:** Notations and some characteristics of the parent H-ZSM-5 zeolites.

Notation	Supplier	SiO_2_/Al_2_O_3_	S_BET_, m^2^ g^−1^
HZ23	Zeolist	23	386
HZ27	Degussa	26.8	399

**Table 2:** Notations and some characteristics of the Cu/ZSM-5 samples.

Notation	Synthesis Conditions	S_BET_, m^2^ g^−1^	Cu, wt. %	Cu/Al
9.7CuZ23(SE)	vacuum, 300 °C	318	9.71	1.25
6.4CuZ27(SE)	He flow, 750 °C	300	6.37	0.99
3.1CuZ23(AE)	0.02 M solution, 50 °C	342	3.10	0.45
2.3CuZ23(AE)	0.01 M solution, 50 °C	371	2.30	0.34

**Table 3:** CO adsorption capacity of the Cu/ZSM-5 samples.

Sample	Cu/Al	Desorbed CO, mL g^−1^	CO/Cu
9.7CuZ23(SE)	1.25	20.1	0.55
6.4CuZ27(SE)	0.99	12.5	0.52
3.1CuZ23(AE)	0.45	10.7	0.90
2.3CuZ23(AE)	0.34	6.5	0.74

**Table 4:** Data from CO breakthrough measurements for different Cu/ZSM-5 samples. The capacity at full saturation is denoted by CO_ads_^e^ and the working capacity, by CO_ads_^b^.

Sample	Pre-Treatment	CO_ads_^e^, mL g^−1^	CO_ads_^b^, mL g^−1^	Efficiency, %	t_b_, min	HUB, mm
3.1CuZ23(AE)	O_2_/Ar at 500 °C	1.4	0.0	0	0	5.0
3.1CuZ23(AE)	CO/Ar at 300 °C	9.8	3.9	40	12	3.0
6.4CuZ27(SE)	Ar at 500 °C	13.8	6.0	43	19	2.9
9.7CuZ23(SE)	Ar at 500 °C	25.8	11.0	43	45	2.9

## Supplementary Materials

The following are available online, Table S1: Data on the chemical composition of the samples obtained by ICP and photometric analysis, Table S2: H_2_ consumption in the TPR experiments expressed as H_2_:Cu molar ratio with differently pretreated 3.1CuZ23(AE), Table S3: H_2_ consumption in the TPR experiments expressed as H_2_:Cu molar ratio with differently pretreated 9.7CuZ23(SE), Figure S1: XRF spectra of 6.4CuZ27(SE), Figure S2: H_2_-TPR profiles of 2.3CuZ23(AE), Figure S3: H_2_-TPR profiles of 6.4CuZ27(SE), Figure S4: CO-TPD curves of 6.4CuZ27(SE), Figure S5: CO-TPD curves obtained after CO adsorption at different temperatures on 9.7CuZ23(SE), Figure S6: Breakthrough curves of CO on 6.4CuZ27(SE) at different conditions, Figure S7: Breakthrough curves of CO on 6.4CuZ27(SE) at different humidity, Figure S8: Breakthrough curves of CO on differently pre-treated 3.1CuZ23(AE), Figure S9: Breakthrough curves of CO on 6.4CuZ27(SE) and 9.7CuZ23(SE), Figure S10: Subsequent cycles of adsorption/desorption of CO on 9.7CuZ27(SE).

## Sample Availability

The starting zeolitic materials are commercially available.
