# Supplementary material for: Purification of Hydrogen from CO with Cu/ZSM-5 Adsorbents
Source: Molecules. 2021 Dec 24;27(1):96. doi: 10.3390/molecules27010096 (PMC8746636; doi:10.3390/molecules27010096)
Supplement: Supplementary file 1 [file molecules-27-00096-s001.zip › molecules-1498335-supplementary.pdf]

molecules

MDPI

Purification of Hydrogen from CO with Cu/ZSM-5 Adsorbents

SUPPORTING INFORMATION

Mihail Mihaylov*, Elena Ivanova, Videlina Zdravkova, Stanislava Andonova, Nikola Drenchev, Kristina Chakarova, Radoslav Kefirov, Rositsa Kukeva, Radstina Stoyanova, and Konstantin Hadjiivanov*

Institute of General and Inorganic Chemistry, Bulgarian Academy of Sciences, Sofia 1113, Bulgaria

*Correspondence: MM, misho@svr.igic.bas.bg; KH, kih@svr.igic.bas.bg

Table S1. Data on the chemical composition of the samples obtained by ICP and photometric analysis.

№	Sample	Al, wt%	Cu, wt%	Cl, wt%
1	6.4CuZ27(SE)	2.74	6.37	1.35
2	9.7CuZ23(SE)	2.95	9.71	1.50
3	3.1CuZ23(AE)	2,92	3,10	-
4	2.3CuZ23(AE)	2.89	2.30	-

Table S2. H_2 consumption in the TPR experiments expressed as H_2:Cu molar ratio with differently pretreated 3.1CuZ23(AE).

Peaks	Pretreated in O_2/He	Reduced in CO/He
Peak 1 (193 – 276 °C)	0.51	0.07
Peaks 2 and 3 (383 – 428 °C)	0.46	0.42
Total	0.97	0.49

Table S3. H_2 consumption in the TPR experiments expressed as H_2:Cu molar ratio with differently pretreated 9.7CuZ23(SE).

Peaks	Pretreated in O_2/He	Reduced in CO/He
Peak 1 (204 – 236 °C)	0.10	0.03
Peaks 2 and 3 (318 – 393 °C)	0.57	0.57
Total	0.67	0.60

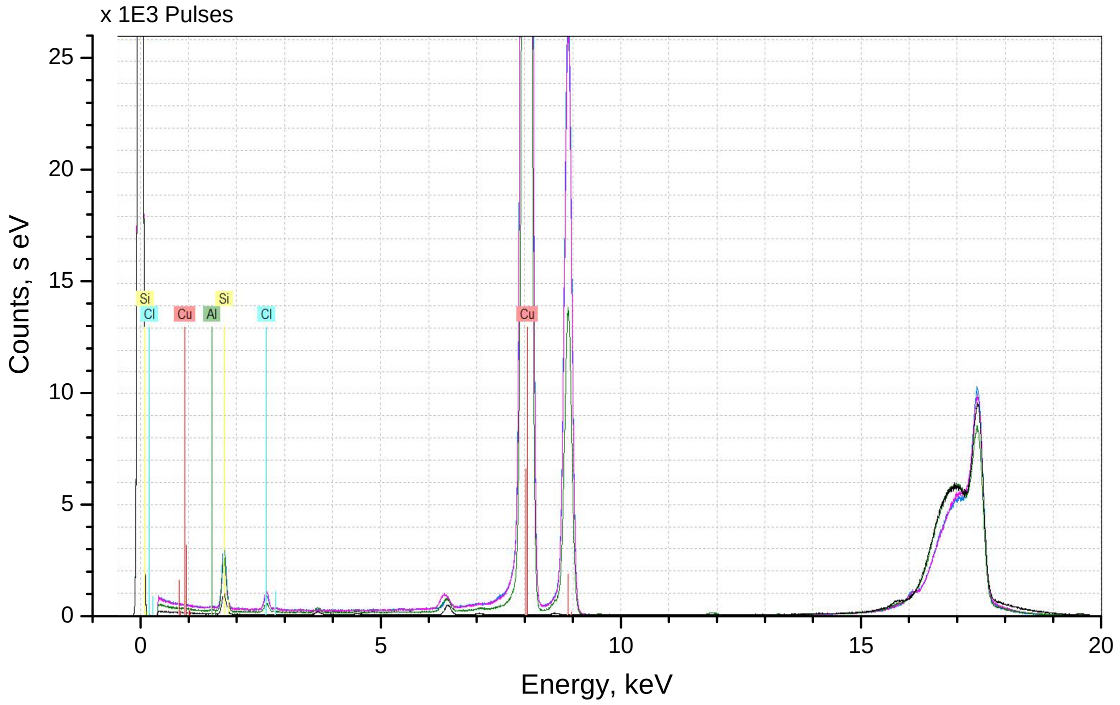

Figure S1. XRF spectra of 6.4CuZ27(SE).

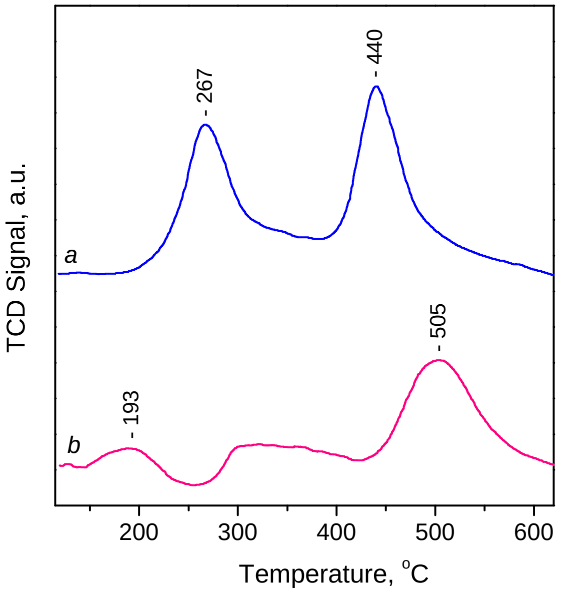

Figure S2. H_2-TPR profiles of 2.3CuZ23(AE): pretreated in He/O_2 at 500 °C (a) and reduced with He/CO at 300 °C (b).

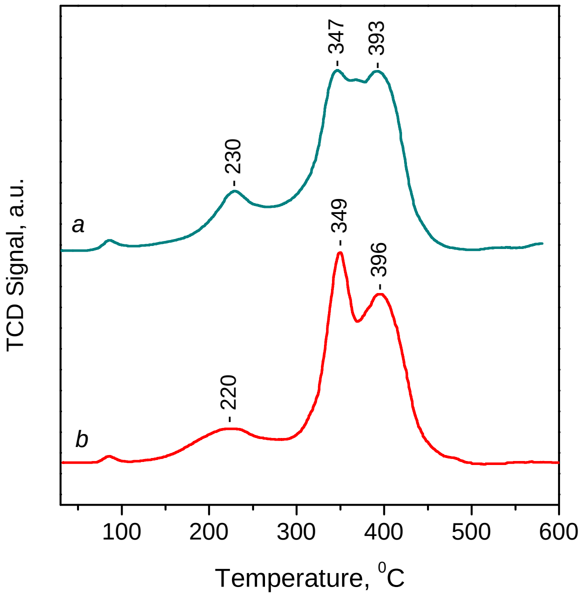

Figure S3. H_2-TPR profiles of 6.4CuZ27(SE): pretreated in He at 500 °C (a) and reduced with He/CO at 300 °C(b).

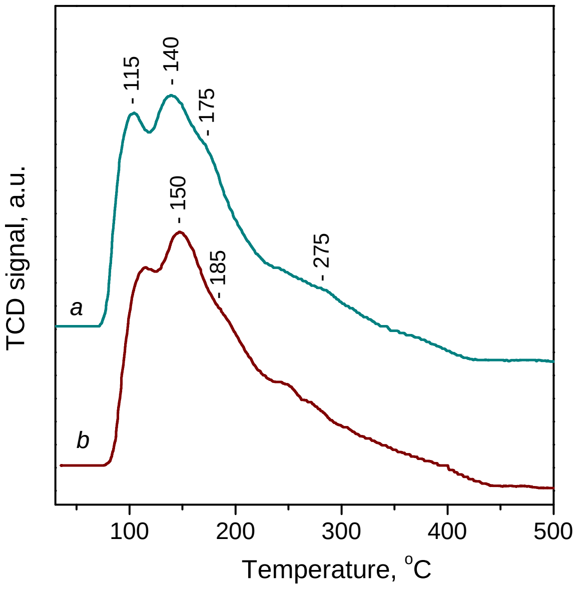

Figure S4. CO-TPD curves of sample 6.4CuZ27(SE) activated in He (a) and reduced with CO (b).

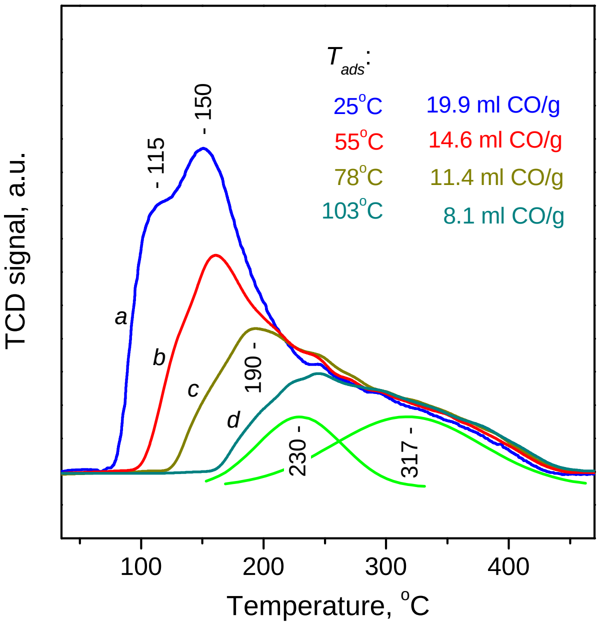

Figure S5. CO-TPD curves obtained after CO adsorption on 9.7CuZ23(SE). The applied T_{ads}: 25 (a), 55 (b), 78 (c) and 103 °C.

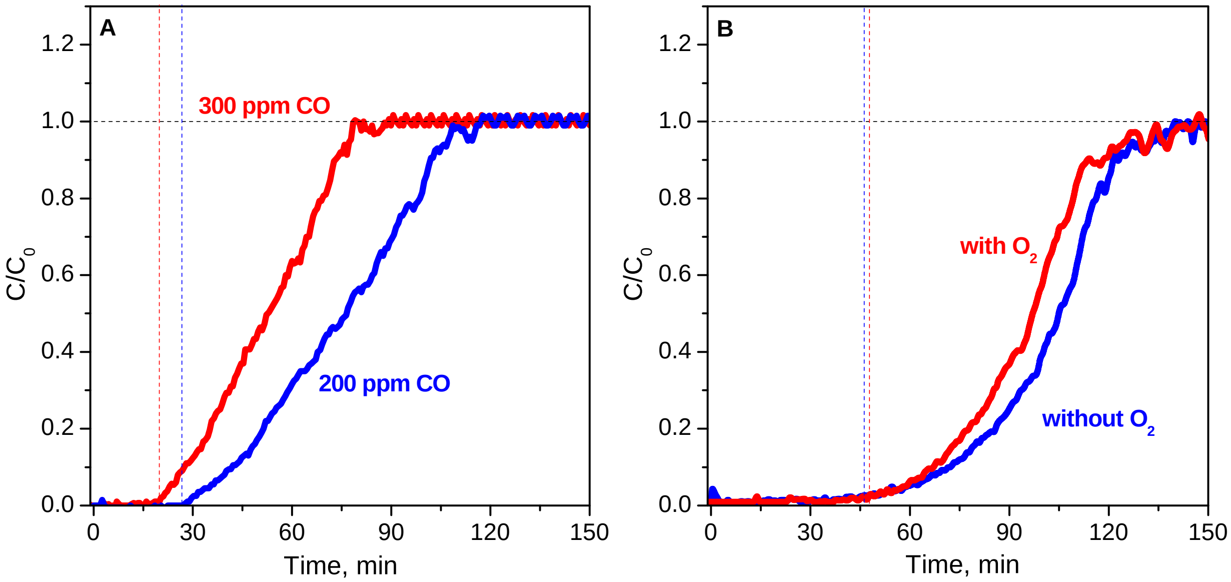

Figure S6. (A) Breakthrough curves of CO on 6.4CuZ27(SE) at different feed gas concentrations of CO, 200 or 300 ppm. (B) Breakthrough curve of CO on 9.7CuZ23(SE), in the absence and presence of 700 ppm O_2.

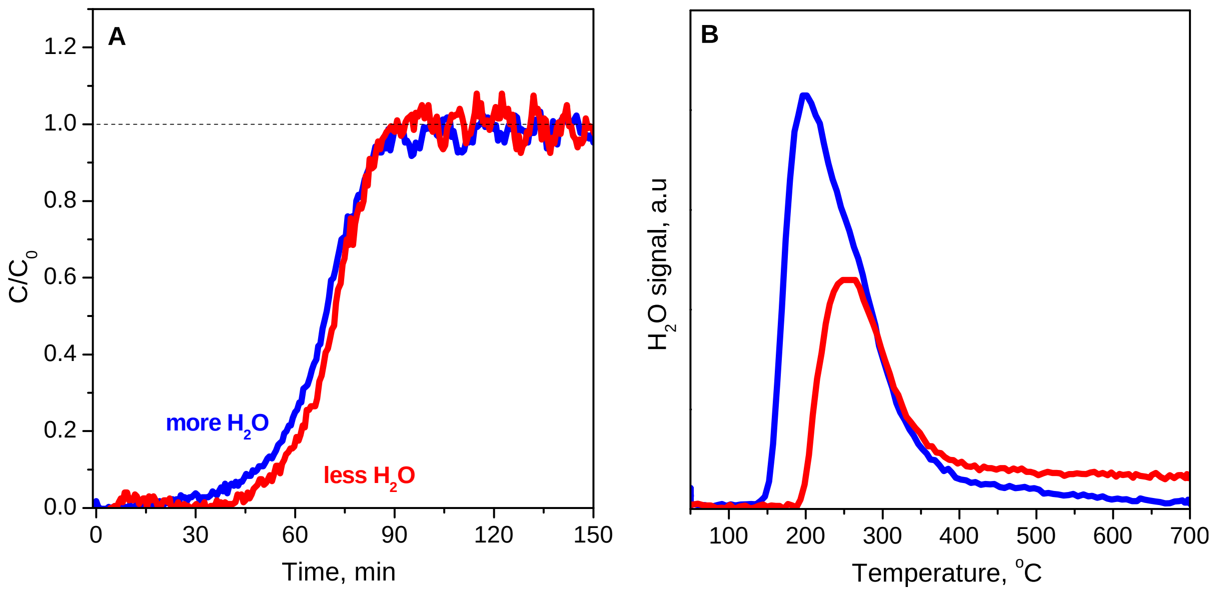

Figure S7. (A) Breakthrough curves of CO on 6.4CuZ27(SE) at different humidity. (B) TPD of water in both caces.

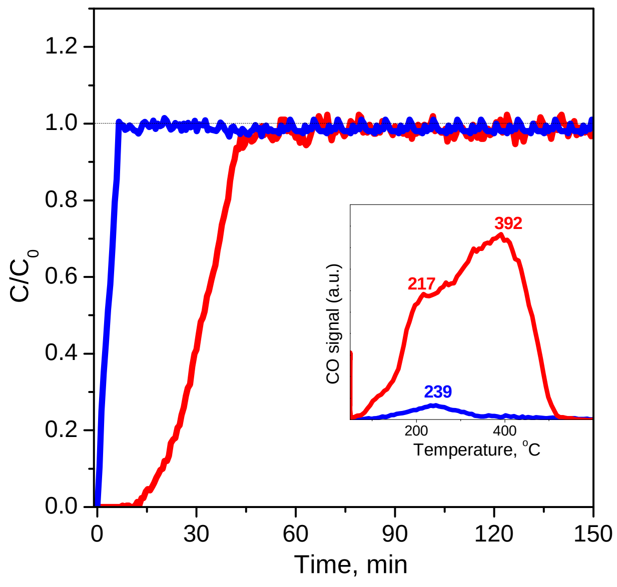

Figure S8. Breakthrough curves of CO on 3.1CuZ23(AE), pre-activated in O_2/Ar (blue) or reduced with CO/Ar (red). The inset shows the respective CO-TPD curves.

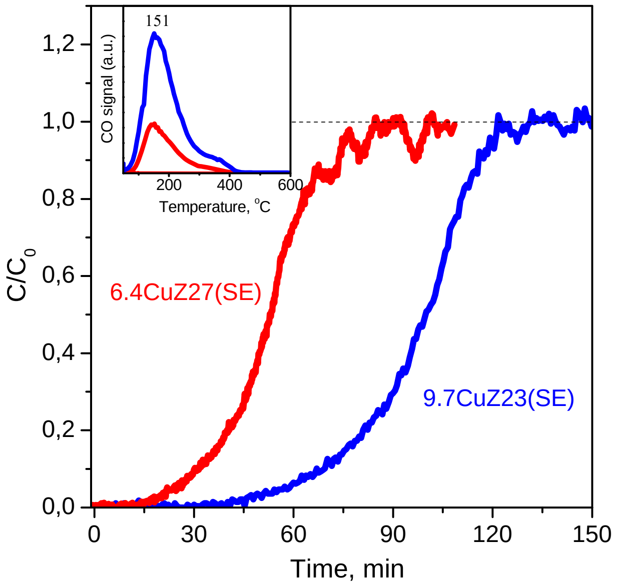

Figure S9. Breakthrough curves of CO on 6.4CuZ27(SE) (red) and 9.7CuZ23(SE) (blue) preactivated in Ar. The inset shows the respective CO-TPD curves.

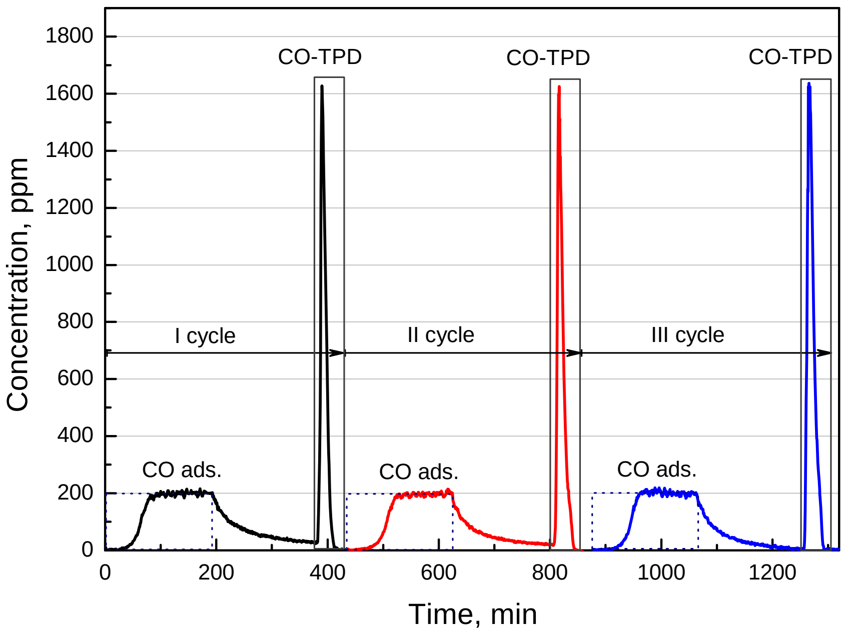

Figure S10. Subsequnt cycles of adsorption (breakthrough curve) and desorption (TPD curve) of CO on 9.7CuZ27(SE).
